# Acoustic Stimulation by Shunt-Diode Pre-Linearizer Using Very High Frequency Piezoelectric Transducer for Cancer Therapeutics

**DOI:** 10.3390/s19020357

**Published:** 2019-01-16

**Authors:** Hojong Choi, Se-woon Choe

**Affiliations:** Department of Medical IT Convergence Engineering, Kumoh National Institute of Technology, Gumi 39253, Korea; hojongch@kumoh.ac.kr

**Keywords:** very high-frequency piezoelectric transducer, pulse-echo detection, shut-diode pre-linearizer, acoustic stimulation

## Abstract

In this paper, we proposed cancer cell acoustic stimulation by shunt-diode pre-linearizer scheme using a very high frequency (≥100 MHz) piezoelectric transducer. To verify the concept of our proposed scheme, we performed pulse-echo detection, and accessed therapeutic effects of human cervical cancer cells exposed to acoustic stimulation experiments using 100 MHz focused piezoelectric transducer triggered by PA with and without the proposed shunt-diode pre-linearizer scheme. In the pulse-echo detection responses, the peak-to-peak voltage of the echo signal when using the PA with shunt-diode pre-linearizer (49.79 mV) was higher than that when using the PA alone (29.87 mV). In the experimental results, the cell densities of cancer cells on Day 4 when using no acoustic stimulation (control group), the very high-frequency piezoelectric transducer triggered by PA only and PA combined with proposed pre-linearizer schemes (1 V and 5 V DC bias voltages) showed 100%, 92.8 ± 4.2%, 84.2 ± 4.6%, and 78 ± 2.9%, respectively. Therefore, we confirmed that the shunt-diode pre-linearizer could improve the performances of the pulse signals of the PA, thus, enabling better therapeutic stimulation performances for cancer cell suppression.

## 1. Introduction

Ultrasound systems have been widely used in a variety of imaging, acoustic stimulation, and therapy applications [1,2,3]. Therapeutic ultrasound is usually applied to the destruction of primary solid tumors and metastatic disease with varied operating conditions including intensity, frequency, pulse repetition frequency, pulse duration, and the exposure time [4]. Various cancer therapeutic treatments using ultrasound have been used to damage tumor, or cancer cells, such as sonodynamic therapy, ultrasound-mediated chemotherapy, sonoporation, gene therapy, and anti-vascular ultrasound therapy and related studies have included in vitro and in vivo experiments [5]. Either structural or molecular damage at the tumor or cancer cell membrane produced by ultrasound stimulation may cause almost complete necrosis of the cells [6]. Consequently, the interaction of ultrasound stimulation with cancerous tissues or solid tumor cells and the subsequent biological alterations have been intensively examined over the past five decades [6,7,8]. A number of literatures have described effects that show significant potential to lead to cell death by the use of ultrasound alone or the combination of chemotherapy and ultrasound [9,10,11]. The ultrasound systems are composed of transmitters, piezoelectric transducers, and receivers [12,13]. The power amplifier (PA) is the most critical electronic component in the transmitters because the PA directly powers the piezoelectric transducers, generating acoustic signals which are delivered into the desired targets [14,15]. The piezoelectric transducer is also the main component to critically influence the signal quality in the ultrasound system [16]. Therefore, PA performance has been a critical issue which affects acoustic stimulation performance [15]. The most important performance parameters of the PA are power gain, and bandwidth for piezoelectric transducer applications since these parameters are related to the sensitivity and resolution of the ultrasound systems, respectively [2]. The sensitivity of the piezoelectric transducers could be affected by the PA performance because transmitted signals generated from a PA induces the piezoelectric transducers [14,15]. Therefore, the design of the PA to attain power gains is an important issue. In wireless or mobile systems, pre-linearizer schemes have been utilized to improve the linearity or reduce power gain deviation of the PA because of the limited frequency ranges for each different industry applications [17,18].

In the piezoelectric transducer applications, the pre-linearizer techniques combined with ultrasound electronic circuits or systems have been recently introduced to reduce the high-order harmonic distortions of low frequency (≤15 MHz) piezoelectric transducers for ultrasound imaging applications [19]. For low-frequency ultrasound therapy applications, contrast agents are another method which is widely used to improve the performances such that they facilitate sonoporation, thus, enhancing therapeutic effects [20,21,22]. Sonoporation is also another method to improve the cell permeability such that it enhances the therapeutic agent’s penetration into the cells [23]. In ultrasound systems, lower power gain reduces the penetration depth of the target and increases the loss of acoustic energy in the target area [24,25,26].

**Figure 1:**
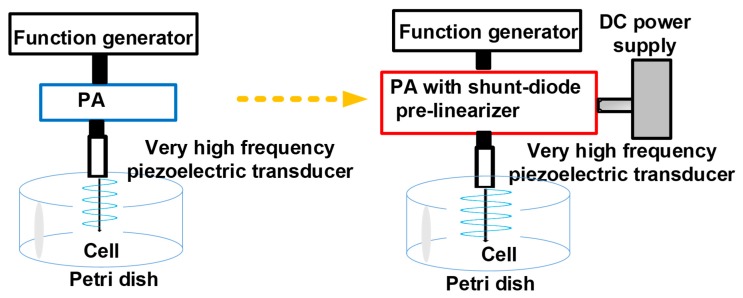
Concept of the acoustic stimulation when using the isolated power amplifier (PA) or PA with shunt-diode pre-linearizer.

Our developed pre-linearizer electronic device has been proposed to reduce maximum power gain deviation points of the PAs for higher power levels, thus, improving the sensitivity of the piezoelectric transducers. Therefore, we believe that reducing power gain deviations using a PA with a pre-linearizer circuit could affect cancer cell suppression. In our best knowledge, we are the first to propose the concept that pre-linearized PAs for very high frequency (≥100 MHz) piezoelectric transducers could affect cancer cell proliferation since there are few researches about cancer cell proliferation using very high-frequency piezoelectric transducer applications [27]. To prove this concept, we performed pulse-echo detection using a very high-frequency piezoelectric transducer to find the suitable stimulation conditions of the shunt-diode pre-linearizer. Afterwards, an isolated PA and a PA with a shunt-diode pre-linearizer circuit combined with a very high-frequency piezoelectric transducer were used for cancer cell viability experiments as described in Figure 1.

Especially for very high-frequency piezoelectric transducers, small size tumor cell (<1 cm) treatment could be possible due to the small beam focus of the piezoelectric transducer (<1 cm) [27]. However, very high-frequency piezoelectric transducers produce relatively low-level sensitivity and low detection level compared to low-frequency piezoelectric transducers [13]. In addition, the stable design of the PA working at very high frequency is challenging because high voltage operation and parasitic impedances could increase operating temperatures of the main transistors of PA, deteriorating the power gain deviation of the PA which can directly affect very high-frequency piezoelectric transducer performance [16,17]. Therefore, we developed the shunt-diode pre-linearizer to improve the performance of the PAs. The paper is organized as follows: Section 2 covers the architecture of the PA with the shunt-diode pre-linearizer and the preparation of the cancer cell experiment. Section 3 covers the pulse-echo detection results of the PA with and without the shunt-diode pre-linearizer and the cancer cell experimental results of the cancer cell when using a very high-frequency piezoelectric transducer triggered by PA with and without the shunt-diode pre-linearizer. Section 4 concludes of the article.

## 2. Materials and Methods

**Figure 2:**
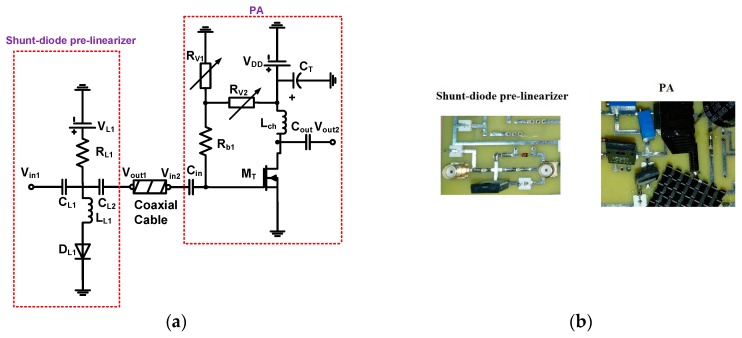

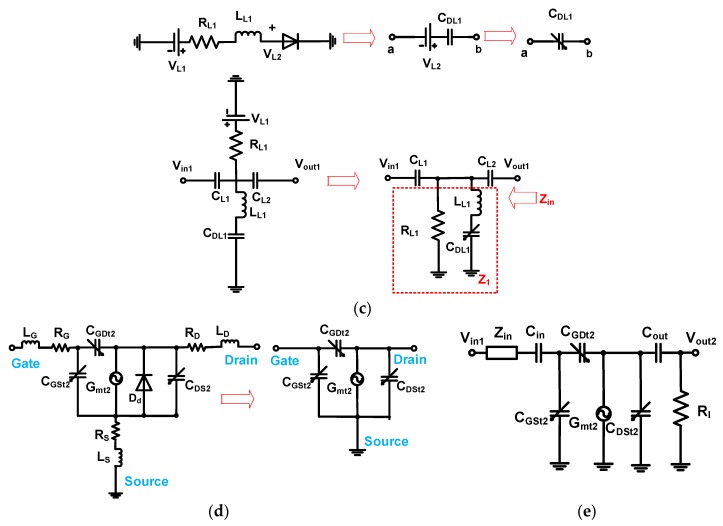
(**a**) Schematic diagrams and (**b**) implemented PA with shunt-diode pre-linearizer; the equivalent circuit models of the (**c**) diode and shunt-diode pre-linearizer, (**d**) large signal non-linear power MOSFET (metal-oxide-semiconductor field-effect transistor), and (**e**) PA with shunt-diode pre-linearizer.

The shunt-diode pre-linearizer and PA were implemented on the fabricated two-layer printed circuit board. A 10 cm, 50 Ω coaxial cable was used to be connected between the shunt-diode pre-linearizer and PA. To avoid the performance degradation, we used very short and small distance copper lines, less than 1 cm, to be connected between the electronic components. In addition, the signal power lines from the input port to the output port did not cross over the ground planes to avoid possible performance degradation. Figure 2a shows the implemented shunt-diode pre-linearizer and PA. The schematic diagrams of the shunt-diode pre-linearizer and PA are shown in Figure 2b. To guarantee stable working ranges of the PA design for long period therapeutic treatments, we also need to consider the worst-case scenarios for PA components, such as high temperature and long hours of operation. Therefore, a 2-cm square feet heat-sink (Aavid Thermalloy, San Jose, CA, USA) attached to the top and bottom of the primary transistor was used to minimize performance suppression due to the sudden temperature variations. The surface mounted device components can be used for the pre-linearizer. However, the PA is a typical Class A-type PA working in high-voltage or high-power environments such that power resistors, a choke inductor, and an electrolytic capacitor must be used because surface mount devices cannot be tolerated under high-voltage or high-power environments. The wideband bias choke inductor was working at high frequency and in a high current environment. Therefore, we typically consider the high frequency and high voltage environment for the PA design. The electrolytic capacitor (C_T_) was used to filter out possible low-frequency noise from the power supply (V_DD_). A large value power resistor and inductor were used to minimize the DC voltage drop such that the maximum voltage amplification could be satisfied in the PA. A variable resistors (R_V1_ and R_V2_, Panasonic electronics Inc., Newark, NJ, USA) were used to find the proper DC bias voltages of the PA. Even though these two capacitors generate the gain suppressions and harmonic distortions, two DC bias capacitors should be used in the PA because high voltage DC can deteriorate the performance of the ultrasonic transducers. The high pass filter which consists of the capacitor and the inductor (L_L1_, Coilcraft Inc., Silver Lake Road, IL, USA) in the shunt-diode pre-linearizer block the unwanted low-frequency AC signal from the input (V_in1_). The inductor (L_L1_) also needs to be used to be compensated with capacitances for the PA [28].

The diode current can be represented as followed by [29]

(1) $$\begin{array}{l} I_{D}=I_{S}\left[\exp\left(\frac{{\mathrm{qV}}_{L2}}{\mathrm{kT}}\right)-1\right]\\ \;V_{L2}=V_{L1}-I_{D}R_{L1} \end{array}$$

where I_s_ is saturation current of the diode.

Since the parasitic resistance is very small, the diffusion capacitance and junction capacitance for the forward bias condition remains [29]. Therefore, the diode capacitance (C_DL1_) is composed of the diffusion and junction capacitances. As shown in Figure 2c, the actual bias voltage of the diode (V_L2_) was subtracted by the current from the voltage source (V_L1_) if the bias resistor (R_L1_) is considered. As shown in Figure 2c, the diode equivalent circuit model is simplified when the forward bias voltage (V_L1_) is applied. To consider the non-linear effect due to the diode current effect, the variable diode capacitance symbol (C_DL1_) was used for the equivalent circuit analysis of the shunt-diode pre-linearizer. As shown in Figure 2c, the shunt-diode pre-linearizer is composed of only passive electronic components such that it may reduce the gain of the PA at certain frequency ranges. This could be an issue because the higher power of the PA needs to be transmitted to the very high-frequency piezoelectric transducer due to very low sensitivity [30]. As shown in Figure 2c, the parallel impedance of the shunt-diode pre-linearizer (*Z_1_*) is represented as followed by

(2) $$Z_{1}=R_{L1}//\left({\mathrm{sL}}_{L1}+\frac{1}{{\mathrm{sC}}_{\mathrm{DL}1}}\right)=\frac{1}{\frac{1}{R_{L1}}+\frac{1}{{\mathrm{sL}}_{L1}+\frac{1}{{\mathrm{sC}}_{\mathrm{DL}1}}}}$$

The impedance of the shunt-diode pre-linearizer (*Z_in_*) is then, represented as followed by

(3) $$Z_{\mathrm{in}}=\frac{1}{{\mathrm{sC}}_{L1}+\frac{1}{{\mathrm{sL}}_{L1}+\frac{1}{{\mathrm{sC}}_{\mathrm{DL}1}}}+\frac{1}{R_{L1}}}+\frac{1}{{\mathrm{sC}}_{L2}}=\frac{1}{\frac{{\mathrm{sC}}_{L1}R_{L1}+1}{R_{L1}}+\frac{1}{\frac{s^{2}L_{L1}C_{\mathrm{DL}1}+1}{{\mathrm{sC}}_{\mathrm{DL}1}}}}+\frac{1}{{\mathrm{sC}}_{L2}}=\frac{R_{L1}(s^{2}L_{L1}C_{\mathrm{DL}1}+1)}{s^{3}R_{L1}C_{L1}L_{L1}C_{\mathrm{DL}1}+s^{2}L_{L1}C_{\mathrm{DL}1}+{\mathrm{sR}}_{L1}C_{L1}+1+{\mathrm{sR}}_{L1}C_{\mathrm{DL}1}}+\frac{1}{{\mathrm{sC}}_{L2}}$$

If we plug s = j2πf_c_ into the impedance of the shunt-diode pre-linearizer, we obtain the input impedance as followed by

(4) $$Z_{\mathrm{in},s=j2{\pi f}_{c}}=\frac{R_{L1}\left(1-4\pi^{2}f_{c}{}^{2}L_{L1}C_{\mathrm{DL}1}\right)}{1-4\pi^{2}f_{c}{}^{2}\left(1-j2{\pi f}_{c}R_{L1}C_{L1}\right)L_{L1}C_{\mathrm{DL}1}+j2{\pi f}_{c}R_{L1}(C_{L1}+C_{\mathrm{DL}1})}+\frac{1}{j2{\pi f}_{c}C_{L2}}$$

where f_c_ is the operating frequency of the PA.

As described in Equation (4), the diode capacitances (C_DL1_) with the bias resistor (R_L1_), other capacitance (C_L1_, C_L2_), and inductance (L_L1_) could be affected such that those components in input impedance could affect the power gain of the PA with shunt-diode pre-linearizer. As shown in Figure 2d, the large signal non-linear power MOSFET model could be simplified because the parasitic resistances (R_G_, R_D_, and R_S_) and inductances (L_G_, L_D_, and L_S_) could be neglected [31,32]. The drain-source capacitance (C_DS2_) are included with parasitic diode capacitance of the power MOSFET model. In the large signal non-linear power MOSFET model, the capacitances have the non-linear behavior in certain voltage levels, so the capacitance values of C_GSt2_, C_GDt2_, and C_DSt2_ are supposed to be varied depending on the applied gate-source and gate-drain voltages [33]. Therefore, we tried to analyze the impedances of the power amplifier with pre-linearizer. In the equivalent circuit model of the large signal non-linear power MOSFET, we put the arrow marks for the capacitances due to non-linear capacitance effects caused by the applied voltage. As shown in Figure 2e, the output voltage of the PA with shunt-diode pre-linearizer can be expressed by

(5) $$\begin{array}{c} V_{\mathrm{out}2}=-G_{\mathrm{mt}2}\cdot V_{\mathrm{in}1}\cdot \left\{Z_{\mathrm{in}}+\frac{1}{j2{\pi f}_{c}(C_{i2}+(C_{o2})}//R_{\mathrm{load}}\right\}\\ =-G_{\mathrm{mt}2}\cdot V_{\mathrm{in}1}\cdot \left\{\frac{R_{L1}\left\{1-4\pi^{2}f_{c}{}^{2}L_{L1}C_{\mathrm{DL}1}\right\}}{1-4\pi^{2}f_{c}{}^{2}\left(1-j2{\pi f}_{c}R_{L1}C_{L1}\right)L_{L1}C_{\mathrm{DL}1}+j2{\pi f}_{c}R_{L1}(C_{L1}+C_{\mathrm{DL}1})}+\frac{1}{j2{\pi f}_{c}C_{L2}}+\frac{1}{j2{\pi f}_{c}(C_{i2}+C_{o2})+\frac{1}{R_{\mathrm{load}}}}\right\} \end{array}$$

where G_mt2_ is the transconductance of the primary transistor (M_T_), C_i2_ is the combined input, gate-source, and gate-drain parasitic capacitances (C_in,_, C_GSt2_, and C_GDt2_) of the primary transistor (M_T_), and C_o2_ is the combined drain-source of the primary transistor (M_T_) and output capacitance (C_out_), and R_load_ is the load resistance.

As shown in Equation (5), the impedances of the shunt-diode pre-linearizer affect the output of the PA. Therefore, we can expect that the signal might be amplified with signal loss, thereby driving unwanted power gain deviations of the PA. However, the equations for the impedance and output of the PA with a shunt-diode pre-linearizer is complex to predict the circuit behaviors when we consider the effects of capacitance and inductance values at certain voltages or powers. Additionally, the simulation data vs. voltages or powers for power MOSFET are inaccurate [34]. Therefore, we need to measure the powers of the PA with shunt-diode pre-linearizer at certain power levels for further analysis. In the next section, we will measure the power gain deviation with respect to the DC bias voltage in the shunt-diode pre-linearizer with PA.

To evaluate the therapeutic effects of the proposed linearizer, the proliferation suppressing ratios (PSRs) was measured on the human cervical cancer cell (HeLa, Korean Cell Line Bank, Seoul, Korea) *in vitro*. HeLa cells were cultured in a high-glucose Dulbecco’s Modified Eagle Medium containing 10% Fetal Bovine Serum with 1% penicillin streptomycin. The prepared cells were incubated at 37 °C in a humidified incubator with 5% CO_2_. They were washed with phosphate-buffered saline to isolate the cells and prepared an approximate concentration of $1\times 10^{6}$ cells/mL. Next, appropriate cells were cultured in the petri dish and then, proposed ultrasonic stimulation was generated and consequently counted as Day 0. To stimulate the same spot on the prepared culture dish, an ultrasound transducer clamper printed using a commercial 3D printer (Cubicon 3DP-310F, Cubicon Inc., Seoul, Korea) was used. It was positioned and retained on the same surface of the growth media until Day 4. All prepared samples were divided into four groups: control group (no ultrasonic induction, *n* = 5), PA group (ultrasonic induction with PA, *n* = 5), PA with shunt-diode pre-linearizer 1 V (when 1 V DC bias voltage was applied, *n* = 5), and PA with series-diode linearizer 5 V (when 5 V DC bias voltage was applied, *n* = 5). The ultrasound signal was induced for about 30 min for four consecutive days. The brightfield images of the ultrasound signal-focused area in the petri dish were taken instantly after ultrasound signal induction by an inverted fluorescent microscope (IX73 with a DP80 camera, Olympus, Japan). Numerous image processing techniques were applied to isolate the quantitative characteristics of each group using the MATLAB software (MathWorks, Natick, MA, USA). The PSR for an individual group was computed by dividing the difference of cell density of the experimental group and the control group by the cell density of the control group on Day 4.

## 3. Results and Discussion

### 3.1. Experimental Performance Verification of the PA with Shunt-diode Pre-linearizer

**Figure 3:**
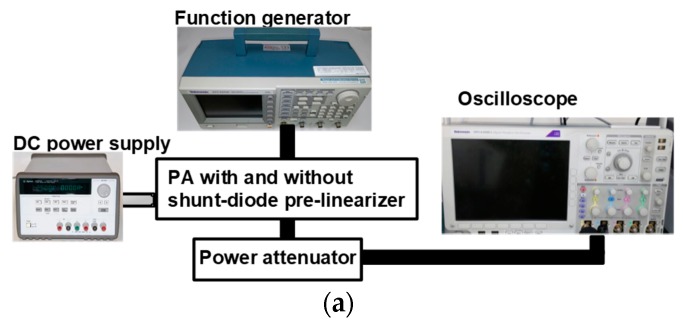

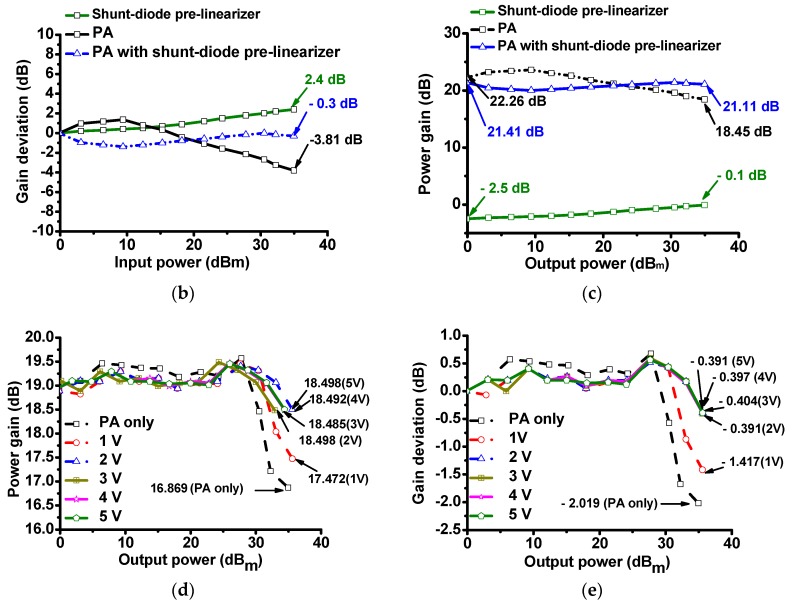
(**a**) The experimental setup of the PA with and without shunt-diode pre-linearizer; expected results of (**b**) gain deviation and (**c**) power gain graphs of the PA with and without shunt-diode pre-linearizer at 5 V DC voltage; (**d**) power gain deviation and (**e**) gain deviation graph of the PA with and without shunt-diode pre-linearizer.

Figure 3a shows the experimental setup of power gain deviation of the PA with and without shunt-diode pre-linearizer. The input power signals from the function generator (AFG3252C, Tektronix Inc., Beaverton, OR, USA) were sent to the PA with and without the shunt-diode pre-linearizer under different DC bias conditions. The amplified signals were attenuated by the 40-dB power attenuator (BW-40N100W+, Mini-circuits, Brooklyn, NY, USA) and output powers were displayed on the oscilloscope (MSO4024B, Tektronix Inc., Beaverton, OR, USA) to calculate the power gain deviation of the PA with and without the shunt-diode pre-linearizer. The power gain is the ratio of the output power to the input power of the PA, and the power gain deviation is how much the power gain deviates over the frequency ranges [33]. Therefore, the power gain was calculated by the ratio of the output power amplitude to the input power amplitude of the power amplifier and the power gain deviation was calculated by how much each power gain at the certain frequency ranges was deviated compared to the power gain at the lowest output power. The power attenuator (BW-40N100W+) must be used to reduce the high voltage signal generated from the PA because the maximum input voltage is 5 V in the 50 Ω impedance setting of the oscilloscope (MSO4024B) and the optimal impedance of the ultrasonic transducer is 50 Ω.

Figure 3b,c show the expected results of the gain deviation and power gain of the shunt-diode pre-linearizer, PA only and PA with shunt-diode pre-linearizer at 5 V DC voltage. In the expected results, the gain deviation of the PA decreases while the gain deviation of the shunt-diode pre-linearizer increases as output power increases. Therefore, the gain deviation of the PA with shunt-diode pre-linearizer at large output power decreases much lesser than that at low output power. Figure 3d,e show measured power gain and power gain deviations of the PA with and without the pre-linearizer, respectively. An input burst sine waveform generated from the function generator (AFG3252C) was applied to the PA when 1, 2, 3, 4, and 5V DC voltages were biased to the shunt-diode pre-linearizer since the diode in the pre-linearizer was working over 0.7 V DC. The amplified output signal was then sent to the oscilloscope (MSO4024B) through the power attenuator (BW-40N100W+). The output voltage signal was measured to calculate the power gain and power gain deviation of the PA with and without the shunt-diode pre-linearizer. The simulation data of the power transistor models for PA design were sometimes inaccurate [18,34]. The measured lowest power gain points when using PA with and without shunt-diode pre-linearizer were marked to show the linearity capability of the shunt-diode pre-linearizer for PA. The largest power gain of the PA with shunt-diode pre-linearizer (17.472 dB at 1 V DC) is still higher than that of the PA without shunt-diode pre-linearizer (16.869 dB). In Figure 3e, the largest power gain deviation of the PA with and without shunt-diode pre-linearizer was further marked to compare the linearity value of the PA with and without the pre-linearizer. The largest gain deviation of the PA (−2.019 dB) is still greater than that of the PA with shunt-diode pre-linearizer (−1.417 dB at 1 V DC). The passive electronic components reduced the power gain of the PA. However, the largest output power of the PA was further reduced with the help of the shunt-diode pre-linearizer. Therefore, these measurement results show that the shunt-diode pre-linearizer reduced power gain deviation at higher output power. This phenomenon is desirable for very high-frequency piezoelectric transducer applications due to very low sensitivity and detection level.

### 3.2. Performance Verifications of the Pulse-echo Response

**Figure 4:**
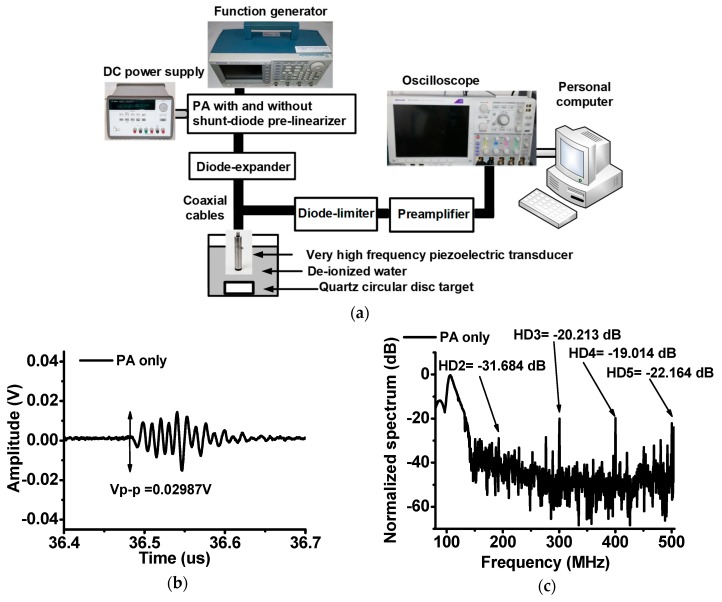

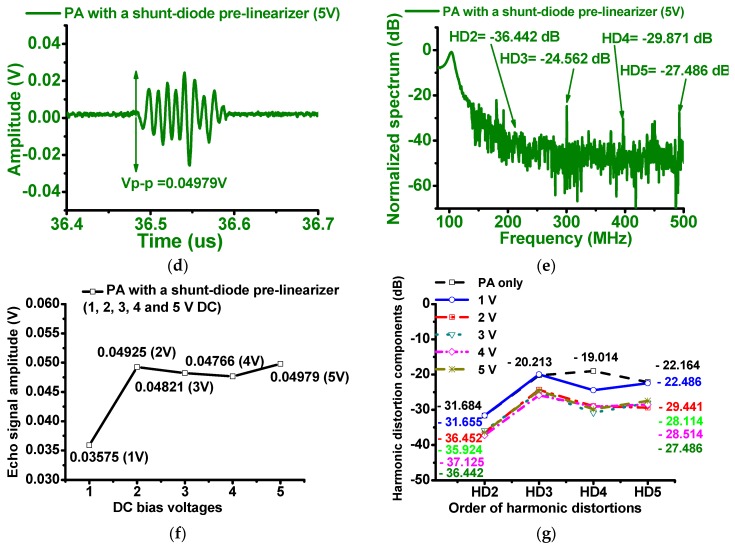
(**a**) Block diagram of the pulse-echo detection setup and its echo data when using the PA with and without shunt-diode pre-linearizer; (**b**) The echo signal and (**c**) its spectrum when 100 MHz input signal applied to the PA without shunt-diode pre-linearizer; (**d**) The echo signal and (**e**) its spectrum when 100 MHz input signal and 1 V DC voltage applied to the PA with shunt-diode pre-linearizer; (**f**) The echo signal amplitudes and (**g**) harmonic distortion components when 100 MHz input signal and 1,2,3,4, and 5 V DC voltage applied to the PA with shunt-diode pre-linearizer.

To verify the capability of the shunt-diode pre-linearizer which can improve the sensitivity of the piezoelectric transducers, we performed typical amplitude-mode pulse-echo detection responses using the very high-frequency piezoelectric transducers as shown in Figure 4a. The amplitude-mode pulse-echo detection responses are typically used to measure the transmit and detection performances of the developed piezoelectric transducer, electronic components, and ultrasound systems [35,36]. The input power generated from the function generators was sent to the PA with and without shunt-diode pre-linearizer and diode-expander (a series cross-coupled diode) and then, amplified power was used to trigger a 100 MHz piezoelectric transducer. The reflected echo signal from the square quartz disc target (2 cm diameter × 1 cm height) was detected by the piezoelectric transducer and then, passed through the diode-limiter (a 50 Ω resistor shunt with a cross-coupled diode) amplified by the preamplifier (AU-1114, MITEQ Inc., Hauppauge, NY, USA). Afterwards, the echo signal was displayed on the oscilloscope (MSO4024B) to calculate the echo spectrum data using a fast Fourier transform algorithm on the personal computer.

In Figure 4b,d, the echo signal amplitude of the PA with shunt-diode pre-linearizer when 5V DC voltage was applied is higher (0.04979 V) than that of the PA without shunt-diode pre-linearizer (0.02987 V). In Figure 4c,e, the echo signal spectrums of the PA with and without shunt-diode pre-linearizer were compared. Especially, with the harmonic imaging techniques in the ultrasound machines it is preferable to use lower harmonic distortions such that we measured several harmonic components in the echo spectrum for the references [14]. The 2nd, 3rd, 4th, and 5th harmonic distortion component (HD2 = −36.442 dB, HD3 = −24.562 dB, HD4 = −29.871 dB, and HD5 = −27.486 dB, respectively) of the PA when 5 V DC bias voltage was applied to the shunt-diode pre-linearizer are lower than that the 2nd, 3rd, 4th, and 5th harmonic distortion component (HD2 = −31.684 dB, HD3 = −20.213 dB, HD4 = −19.014 dB, HD5 = −22.164 dB, respectively) of the PA without shunt-diode pre-linearizer. As shown in Figure 4g, the 2nd, 3rd, 4th, and 5th harmonic distortion component (HD2, HD3, HD4, and HD5) and total harmonic distortion (THDs) values of the PA without shunt-diode pre-linearizer is worse than those values of the PA with shunt-diode pre-linearizer (1, 2, 3, 4, and 5 V DC). For 1 V DC bias voltage for shunt-diode pre-linearizer, the performances of the HDs and THDs are a little bit worse compared to those performances for 2, 3, 4, and 5V DC bias voltage for shunt-diode pre-linearizer because the diode in the shunt-diode pre-linearizer is conducting over 0.7 V DC voltage. Each harmonic distortion was improved by less than 6 dB. However, the echo signal amplitude is improved by about 40% as shown in Figure 4f. Therefore, we can conclude that the PA with shunt-diode pre-linearizer can improve the sensitivity of the very high-frequency piezoelectric transducers. Table 1 summarizes the echo amplitudes, HD2, HD3, HD4, HD5 and THD of the PA without and with shunt-diode pre-linearizer (1, 2, 3, 4, and 5 V DC).

### 3.3. Quantitative Analysis of PSRs for the HeLa Cell Subsequent Acoustic Stimulation

**Figure 5:**
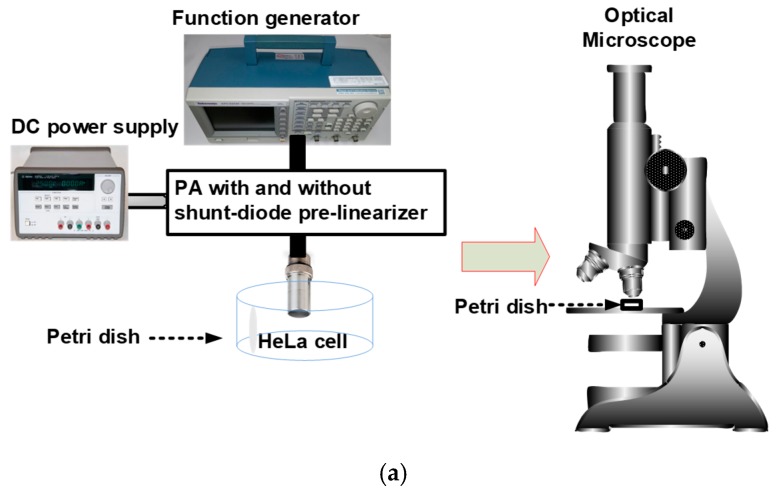

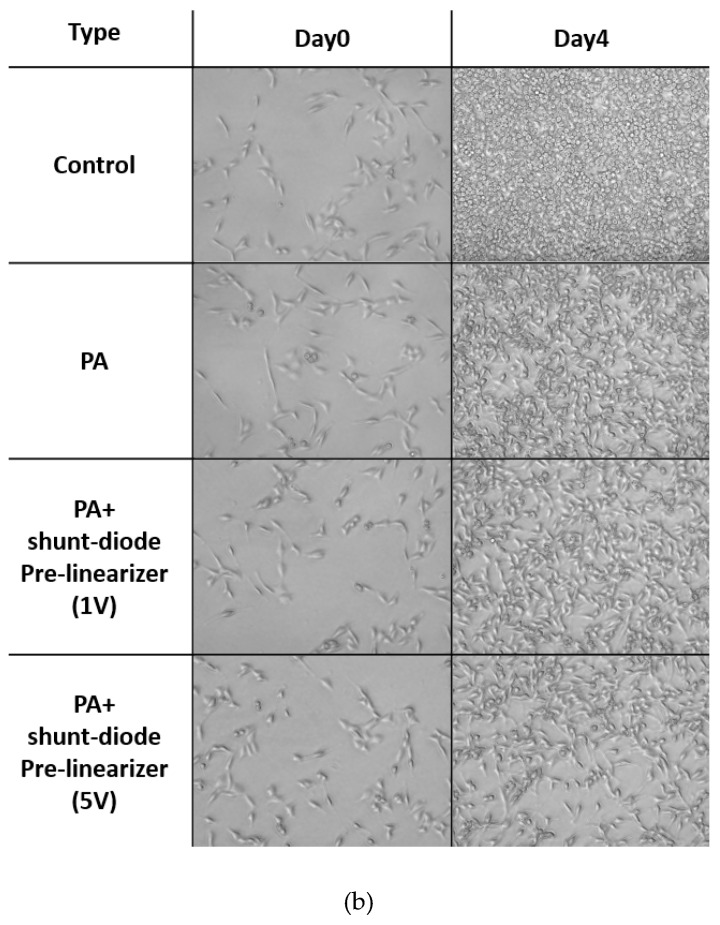
(**a**) Block diagram of the human cervical cancer cell (HeLa) cell experiments using the PA with and without shunt-diode pre-linearizer and (**b**) representative brightfield images of HeLa cell from Day 0 to Day 4 acquired using an inverted microscope.

The shunt-diode pre-linearizer could reduce the power gain-deviation of the PA, thus, increasing the sensitivity or detection capability of the piezoelectric transducers. In the experiment, we will verify the concept that the improved ultrasound signal could suppress the HeLa cell proliferation more effectively. High-frequency ultrasonic stimulus-triggered the process of mechanical effects and acoustic cavitation that causes cell damage to cancer cell necrosis. The process of cavitation can result in structural and/or functional changes including cell lysis, proliferation, synthesis, and migration and one of these alterations can bring about the other changes and vice versa [5]. Since the acoustic stimulus can perform as either an activator or an inhibitor for cell function, the biological effects are determined by a certain acoustic intensity, frequency, and exposure time [37]. Various solid tumor cells have a more inflexible membrane compared to normal cells so that it is easily ruptured by acoustic stimulus. Moreover, free-radicals produced by the ultrasonic cavitation can increase the damage to the extracellular membrane. Therefore, acoustic stimulus can be employed to initiate the structural and functional changes in solid tumor cells and, thus, suppress the cell viability. Figure 5a shows the experimental setup for HeLa cell stimulation using the PA with and without shunt-diode-linearizer. After exposing HeLa cells to the ultrasound signal, the brightfield images were acquired by an inverted microscope as shown in Figure 5b. The image processing techniques were applied using MATLAB software (MathWorks, Natick, MA, USA) to quantify the PSRs by dividing the difference of cell density of the experimental group and the control group by the cell density of the control group on Day 4.

**Figure 6:**
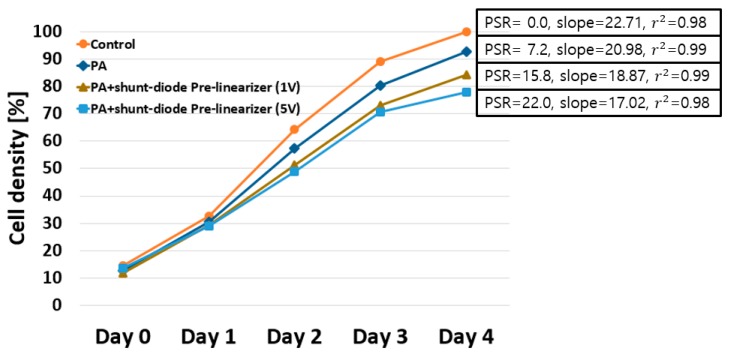
Measured HeLa cell densities when uncovered to the separated ultrasonic PA, PA with shunt-diode pre-linearizers at 1 V and at 5 V DC bias voltages from Day 0 to Day 4. PSR refers to proliferation suppressing ratio compared to the control group on Day 4.

In the experimental results of the echo signals (Figure 4), the PA with pre-linearizer showed different performances at 1 V DC bias and after 1 V DC bias voltage. Therefore, we tested the HeLa cell experiments when using PA only, PA with shunt-diode pre-linearizer at 1 V and 5 V DC bias voltages. Figure 6 shows the computed cell densities and the proliferation suppressing ratios from the acquired brightfield images between the control group, PA only, PA with shunt-diode pre-linearizer at 1 V DC and 5 V DC bias voltages, respectively. Based on the analyzed data, the experimental groups significantly reduce the cell concentrations as compared to the control group. Cell densities stimulated by PA and PA with the shunt-diode pre-linearizer at 1 V and 5 V DC bias voltages were changed to 92.8 ± 4.2%, 84.2 ± 4.6%, and 78.0 ± 2.9%, respectively. Moreover, their PSRs were 7.2%, 15.8%, and 22.0%, respectively. As a result of applying linear regression to the calculated cell densities, outstanding linear correlations (*r*^2^ > 0.98) were attained for each group. Ultrasonic stimulation by the PA with the shunt-diode pre-linearizer at 5 V DC bias voltage showed the most noteworthy PSR value (PSR = 22%, slope = 17.02, *r*^2^ = 0.98) as compared to the control group because this circumstance could improve the power gain of the PA with the shunt-diode pre-linearizer at 1 V and 5 V DC bias voltages, thus, enhancing the echo signal amplitude. Table 2 summarizes the experimental data of HeLa cell densities from Day 0 to Day 4, PSRs compared to the control group on Day 4, and slope with *r*^2^ values by simple linear regression.

## 4. Conclusions

We propose that by using a shunt-diode pre-linearizer improved power gain deviation can be achieved which will increase the sensitivity of the very high frequency (≥100 MHz) piezoelectric transducers, thereby decreasing cancer cell proliferation. To our knowledge, we are the first to propose the concept of the sensitivity performance improvement of very high-frequency piezoelectric transducers with a linearized PA, thus, increasing the suppression of the cancer cell effectively. To verify this concept, we implemented PA with and without shut-diode pre-linearizer on a two-layer printed circuit board and compared the power gain deviation performances of the PA with and without shunt-diode pre-linearizer. In the electronic performance measurement, the largest power gain deviation of the PA with shunt-diode pre-linearizer (18.498 dB at 5 V DC) is higher than that of the PA only (16.869 dB). Afterwards, we performed pulse-echo detection responses and experiment of the acoustic stimulation for cancer cell using very high frequency triggered by PA with and without shunt-diode pre-linearizer. In the pulse-echo detection responses, the peak-to-peak voltage of the PA with shunt-diode pre-linearizer when 5 V DC voltage was applied was higher (0.04979 V) than that of the isolated PA (0.02987 V). According to the results, the most noteworthy changes were observed when HeLa cells were exposed to the ultrasonic stimulation PA with a shunt-diode pre-linearizer when 5 V DC bias voltage was applied (PSR = 22%, slope = 17.02, *r*^2^ = 0.98) as compared to the control group. Therefore, we confirm that the PA with shunt-diode pre-linearizer decreases cancer cell proliferation more effectively when compared to the PA without shunt-diode pre-linearizer.

## Figures and Tables

**Table 1:** Summary of the measured echo signal amplitudes, harmonic distortion (HD2, HD3, HD4, HD5), and total harmonic distortion (THD) of the 100 MHz piezoelectric transducers when using the PA without and with shunt-diode pre-linearizer when 1, 2, 3, 4, and 5 V DC voltages were applied.

	PA only	1 V	2 V	3 V	4 V	5 V
Amplitudes	0.02987	0.03575	0.04925	0.04821	0.04766	0.04979
HD2 (dB)	−31.684	−31.655	−36.452	−35.924	−37.125	−36.442
HD3 (dB)	−20.213	−19.950	−24.331	−25.116	−25.881	−24.562
HD4 (dB)	−19.014	−24.450	−28.988	−30.845	−29.125	−29.871
HD5 (dB)	−22.164	−22.486	−29.441	−28.114	−28.514	−27.486
THD (dB)	−30.806	−33.966	−44.320	−44.561	−45.341	−43.689

**Table 2:** Summary of the numerical experimental data of HeLa cell density results when using PA only, PA with or without shunt-diode pre-linearizer were applied individually.

	Day 0	Day 1	Day 2	Day 3	Day 4	PSR (vs. Control)	Slope	*r* ^2^
Control	14.7 ± 3.8%	32.7 ± 2.6%	64.2 ± 3.8%	89.2 ± 3.7%	100 ± 0.0%	0%	22.71	0.98
PA only	12.8 ± 3.2%	30.6 ± 5.1%	57.4 ± 5.8%	80.4 ± 4.0%	92.8 ± 4.2%	7.2%	20.98	0.99
PA with shunt-diode pre-linearizer at 1V	11.7 ± 1.4%	29.3 ± 1.9%	51.2 ± 4.7%	73 ± 3.4%	84.2 ± 4.6%	15.8%	18.87	0.99
PA with shunt-diode pre-linearizer at 5V	13.7 ± 1.1%	29.1 ± 1.1%	49.0 ± 5.7%	70.8 ± 3.7%	78.0 ± 2.9%	22.0%	17.02	0.98
